# The Impact and Invasive Mechanisms of *Pueraria montana* var. *lobata*, One of the World’s Worst Alien Species

**DOI:** 10.3390/plants12173066

**Published:** 2023-08-26

**Authors:** Hisashi Kato-Noguchi

**Affiliations:** Department of Applied Biological Science, Faculty of Agriculture, Kagawa University, Miki 761-0795, Kagawa, Japan; kato.hisashi@kagawa-u.ac.jp

**Keywords:** adaptation, allelopathy, crop production, economic loss, natural enemy, secondary metabolite, reproduction

## Abstract

*Pueraria montana* var. *lobata* is native to East Asia, and was introduced to many countries due to its potential for multiple uses. This species escaped under the management conditions soon after its introduction, and became a harmful weed species. This species has been listed in the top 100 of the world’s worst invasive alien species. *P. montana* stands expand quickly and threaten the native flora and fauna including microbiota. This species affects the concentration of carbon and nitrogen in soil and aquatic environments, and increases the amount of pollutants in the local atmosphere. Its infestation also causes serious economic losses on forestry and agriculture. Its characteristics of fast growth, thick canopy structure, enormous vegetative reproduction, and adaptative ability to the various environmental conditions may contribute to the invasiveness and naturalization of this species. The characteristics of *P. montana* regarding their defense functions against their natural enemies and pathogens, and allelopathy may also contribute to the invasiveness of this species. Potential allelochemicals such as xanthoxins, *p*-coumaric acid, caffeic acid, methyl caffeate and daidzein, and two isoflavones with anti-virus activity were identified in this species. In addition, fewer herbivore insects were found in the introduced ranges. These characteristics of *P. montana* may be involved in the invasive mechanisms of the species. This is the first review article focusing on the invasive mechanisms of this species.

## 1. Introduction

*Pueraria montana* var. *lobata* (Willd.) Maesen & almedia S.M. Almeida ex Sanjappa & Predeep (Syn. *Pueraria lobata* (Willd.) Ohwi, *Pueraria thunbergiana* (Siebold & Zucc.) Benth., referred to as *Pueraria montana* hereafter), belongs to the Fabaceae family, and is a perennial climbing vine. Yong stems are covered in bronze hairs, and older stems become woody and have a very large diameter, measuring up to 30 cm [1,2,3,4]. This species grows upward through its apex to twine, which is a right-handed spiral, around the stems and branches of trees and other types of vegetation, walls and structures [5,6,7,8,9]. 

**Figure 1:**
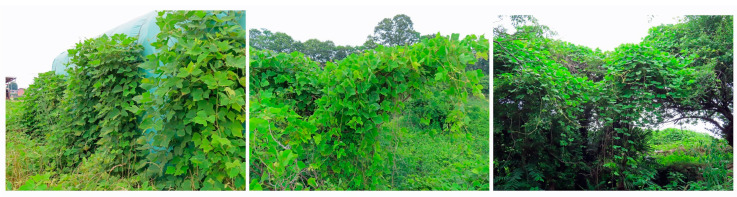
*P. montana* climbing on a greenhouse, trees and other types of vegetation.

**Figure 2:**
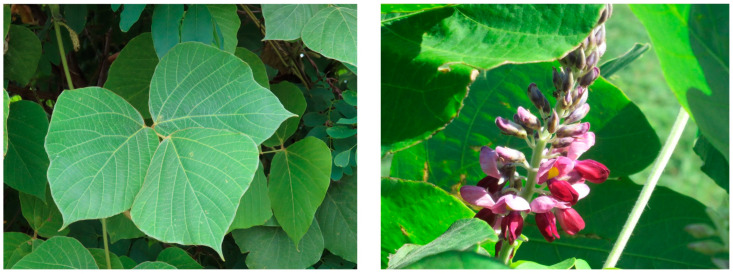
*P. montana*; trifoliate and inflorescence.

Leaves are alternate and large trifoliate, and supported with a hairy petiole (8–20 cm long). The leaflets of the trifoliate leaves are broadly ovate, 2–3 lobed up, 7–20 cm long and 5–20 cm wide [1,2,3,4]. Racemose inflorescences are 10–30 cm long, and the lateral branches on the rachises are greatly reduced and bear 2–3 flowers. The pea-like flowers are 2.5 cm long and contain five violet-purple or reddish-purple petals (one big, two medium and two small sizes) with five fused purple-red sepals. The pistil has an ovary (7–15 cm long) and a globular stigma. There are 10 stamens, of which nine fused and one free. The fruits are hairy pods (4–15 cm in length and 0.5–1.5 cm in width) containing 1–15 kidney-shaped seeds [2,3,4]. This species produces an extensive root system with tuberous roots up to 2 m in length, 50 cm in diameter, and 100 kg in weight, which contain a large amount of starch [2,10] (Figure 1 and Figure 2).

*P. montana* is native to East Asia [3,4,11], and has been introduced to many other countries as an ornamental shade plant, a high-nitrogen forage crop, a control plant for soil erosion, and a plant for manufacturing materials [10,12,13,14,15]. Its roots have been used as a source of starch for traditional foods and medicine in some countries in its native range. Its stalk fiber has also been used for making clothes and fishing nets [10,14,16,17,18]. However, this species grows quickly, escapes from under its management, and replaces previously existing vegetation with its dominant stands [19,20]. Soon after the introduction of the species in North America, the management problems of its overwhelming growth characteristics were realized [10,19]. This species was reported to spread over 3 million hectares of the eastern USA by 2001 [10], and the area covered by the species was estimated to be increasing by 50,000 hectares per year [14]. *P. montana* has been listed as one of the most harmful non-indigenous plant species in USA [21]. It has already spread and naturalized in many other countries in North and South America, Europe, Africa, Australia and South Asia [3,4,11]. This species causes serious negative impacts on the natural ecosystems and agricultural crop production, and has been listed in the top 100 of the world’s worst invasive alien species [3,4,11,22]. There have been some review articles on the introduction history, distribution, impact and management of *P. montana* [1,2]. However, those articles were published 10–20 years ago, and considerable investigations for the species have been carried out since then. In addition, no review article focusing on the invasive mechanism of *P. montana* has been made available. The objective of this review is to discuss the impact and possible invasive mechanisms of *P. montana*. To compile this review article regarding the impacts and invasive mechanisms of the species, the literature has been searched using a combination of the predominant online search engines: Scopus, ScienceDirect and Google Scholar. All possible combinations of *Pueraria montana* with the following words were searched: biology, invasive mechanism, habitat, impact, forestry, agriculture, fire, reproduction, flower, seed, plasticity, adaptation, nutrient, colonization, arbuscular mycorrhizal fugus, rhizobium, endophyte, genetic diversity, allelopathy, allelochemical, allelopathic substance, defense function, natural enemy, pathogen, herbivore, insecticidal activity, fungicidal activity, pharmacology, second metabolite, global warming, management, and biological control. This paper provides an overview of the literature describing the impact and invasive mechanisms of this species.

## 2. Impact of *P. montana*

*P. montana* was reported to threaten native flora, fauna and microbiota in the introduced ranges, and to cause serious economic losses. This section discusses the impact of *P. montana* on aspects of the natural ecosystems such as their species diversity and population, including microbiota, soil nutrients and local atmosphere, and on economic aspects of forestry and agriculture.

### 2.1. Impact on the Natural Ecosystem

**Figure 3:**
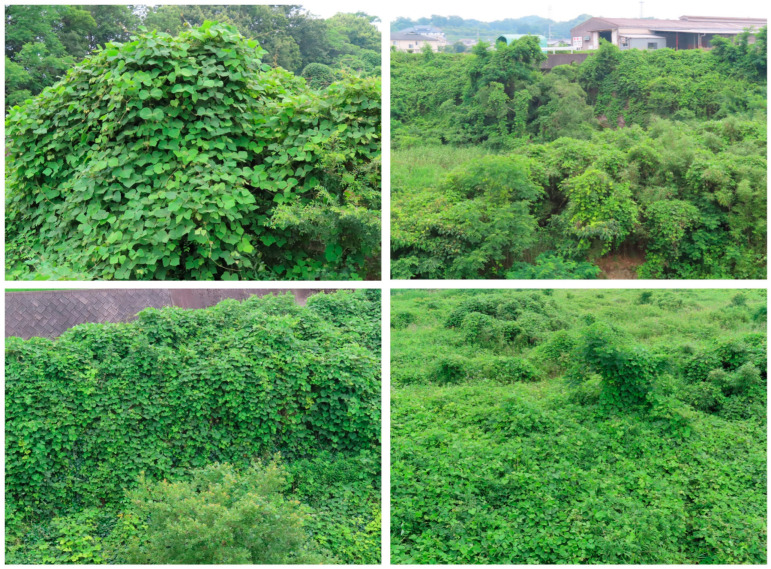
*P. montana* stands covered over trees, a forest, orchard and agricultural field.

*P. montana* is capable of rapid stem elongation and climbing trees via the winding of its stems around the trees, as described in the Introduction and Figure 1. The stems are highly branching and expand their growing space [5,6], and its large trifoliate leaves cover whole trees and occupy forest canopies [1,2,5,6,7]. This species also grows by creeping on other types of vegetation on the ground, and forms dense mats [8]. The thickness of the species mats of well-developed stands is 1.5–2 m [23]. As a result, this species causes complete shading, and disturbs the photosynthesis of the native vegetation, including trees, resulting in the death of indigenous plant species, and eliminating everything in its path [10,12,14,24]. *P. montana* replaced previous existing vegetation such as orchards, plantation crops and young forests [19] (Figure 3).

The number of plant species in *P. montana*-infested forests was about half of that in the neighboring *P. montana*-free forests in Switzerland [23,25]. The *P. montana-*infested forests showed a lower species richness, less understory coverage and a lower density of woody species than its non-infested forests in Mississippi, USA [26]. The density of *P. montana* population was negatively correlated with the diversity and the number of native trees and avian populations in the forests in Tennessee, USA [27]. The reduction in the number and diversity of arthropods and insects has been reported in *P. montana*-infested lands in Europe [22]. *P. montana* also suppressed the forest recovery from storms and other causes of tree fall [1]. This species provides fire ladders to upper-canopy layers, resulting in an increase in the wild fire magnitude and expanses [28].

Plants affect soil carbon concentration through absorption and liberation during the decomposition process of the plant litter [29]. Although plant litter input into the soil under *P. montana*-infested areas increased 22%, carbon concentration in the soil was 28% lower compared to that in soil under its non-infested areas because the carbon-consuming microbial activity was higher in the infested soil than in the non-infested soil [30,31]. A lower carbon concentration may affect the growth of the native plant species.

A high level of arbuscular mycorrhizal fugus colonization was found in the roots of *P. montana* in the native and introduced ranges [32]. Arbuscular mycorrhizal fungi enhance their host plant performance by increasing nutrient and water acquisition, and the defense functions against the pathogen attacks and stress conditions [33,34,35]. More than 90% of plant species associate with arbuscular mycorrhizal fungi [36], and the mutualism of the host plant with arbuscular mycorrhizal fungi is species-specific [37]. *P. montana* altered the species constitution of arbuscular mycorrhizal fungi in the introduced ranges for its own mutualism [32]. The alteration in the arbuscular mycorrhizal fungus constitution may cause the adverse effects of the native plant species.

The *Pueraria* species associates with rhizobia, which belong to the genera *Bradyrhizobium* and *Mesorhizobium* [38,39,40]. The fixing of nitrogen by *P. montana* via symbiosis was estimated to be 22–235 kg per hectare per year [41,42]. Some of the fixed nitrogen is released into the soil during the decomposition process of the *P. montana* residues. The nitrogen concentration in the *P. montana*-infested soil was 2- to 5-fold greater than that of its non-infested soil [43]. The excess nitrogen from the soil is leached into the waterflow, and streams out to rivers, swamps and lakes, affecting the aquatic environment and biodiversity [1,2]. These observations indicate that a large infestation of *P. montana* may alter the nitrogen cycle in the local environments.

The nitrogen (NH_4_^+^) in the soil is also transformed via microbial nitrification (NH_4_^+^ to NO_3_^−^) and denitrification (NO_3_^−^ to N_2_) processes [44,45]. When the overall rate of nitrogen transformation increases, the emission of NO (nitric oxide) and N_2_O (nitrous oxide; powerful greenhouse gas), which are by-products of the nitrification and denitrification processes, tends to increase [46]. The net nitrification and the emission of nitric oxide increased 10- and 2-fold more in the *P. montana-infested* areas than those in the non-infested areas [43,47]. *P. montana* also has the biosynthesis pathway of isoprene, and emits large amounts of isoprene into atmosphere [48,49]. Isoprene affects tropospheric chemistry by forming ozone and smog when nitrogen oxides are coexistent. The isoprene breakdown process also produces peroxyacetyl nitrate and methyl peroxyacetyl nitrate, which are the secondary pollutants in the photochemical smog [50]. Therefore, *P. montana* has considerable potential to affect the local air quality.

These observations indicate that *P. montana* infestation potentially reduces the species diversity and number of native plants, arthropods, insects and birds, and alters the species constitution of the arbuscular mycorrhizal fungi. The thick canopy structure of this species prevents sunlight from penetrating into under-growth vegetation, and disturbs their photosynthesis. This species also affects the concentration of carbon and nitrogen in soil, and the local air quality.

### 2.2. Impact on the Forestry, Agriculture and Others

The productivity loss of the forest industry in USA due to *P. montana* infestation was estimated to be between USD 100 and 500 million per year [1,21]. The management cost of *P. montana* would be USD 500 per hectare per year in pine plantations, thus exceeding the profits for plantations that need a 25-year cultivation [19]. A USD 5.1 billion loss in the Oklahoma timber industry alone due to the infestation was also estimated [51]. The infestation also caused serious changes in the native landscape in national parks, and its control costs were considerable [1]. *P. montana* damages electric cables due to its wrapping around the cables, causing power failures especially in rural areas in the southern USA, and the management costs were estimated to be USD 1.5 million per year [19]. Railway companies also need a considerable amount of capital to manage its infestation along railway lines [1]. 

*P. montana* infestation into agricultural fields caused crop yield loss due to competition, shading and smothering [1]. This species is also involved in the transmission of pathogens to crop plants as a vector for the soybean vein necrosis virus [52], the tobacco ringspot virus [53] and the soybean rust virus [54,55,56]. These pathogenic viruses cause a serious crop yield loss. For example, the infection of soybean rust virus caused a soybean yield loss ranging from USD 240 million to 2 billion in the USA [57]. The soybean yield loss and fungicide costs were estimated to be USD 500 million and 700 million per year in Brazil, respectively [58]. These observations indicate that *P. montana* infestation caused serious economic losses on the industries of forestry, agriculture and others. 

## 3. Possible Mechanisms of the Invasiveness of *P. montana*

*P. montana* was shown to cause serial problems on the natural ecosystems and industry of the introduced ranges as described in the previous section. Although many researchers have investigated the invasive mechanism of the species, no review article has been made available. This section discusses the possible mechanisms of the invasiveness of this species by focusing on the characteristics of its growth, reproduction, adaptation, allelopathy, insecticidal and fungicidal activity, and secondary metabolites.

### 3.1. Growth

*P. montana* grows quickly. Its stem growth rate was 3–20 cm per day, and 20–30 m per single growing season, and reached 63–320 m in length [6,59,60,61]. Frequent branching occurs from the nodes of the main stems and the branches, and the average number of primary and secondary branches was 15 and 18 per main stem, respectively. The total length of stems and branches was up to 360 m per m^2^ [6,61]. The negative effect of the long stem length on the water transport from roots to the apexes and top leaves was compensated for by the transpiration from the large leaves, large stem sapwood areas and water storage, and by the decreasing petiole hydraulic resistance and the maintenance of the high stem hydraulic conductance. These functions support the water transportation of the long stem species [62,63].

The number of leaves of the main stems, primary branches and secondary branches of *P. montana* grown for a single year in pot conditions was 19, 94 and 60, respectively [5], and the leaf area per gram of shoot biomass was 110–150 cm^2^ [1]. This species forms thick canopy layers, and its leaf area was 3.7–7.8 m^2^ per m^2^ of grand area [64], which is a 10- to 15-fold greater leaf area per unit stem compared to that of the mature tree species [1]. This species has also the ability to rapidly reorient its leaves to prevent self-shading, excess solar radiation and high temperature. This movement is caused by the turgor changes induced by the K^+^ flux into the cells of the pulvini at the base of the petioles of the leaves [65,66]. The leaf reorientation and thick canopy of this species enable it to effectively receive solar radiation for the photosynthesis. *P. montana* as a vine utilizes mechanical support by climbing on trees and other vegetations, and minimizes the investment into its own supporting tissues such as its stems and branches. This species allocates large amounts of photosynthetic fixed carbon to the leaves for the production of a great number of large leaves, and to create a thick canopy structure [5,60,67]. The ratio of the carbon allocation to the leaves in *P. montana* was estimated to be 20–28% of the total fixed carbon, whereas it was 1–2% in the mature deciduous tree species [68,69]. *P. montana* is deciduous, and the leaves drop in autumn or early winter [64,70]. The axillary buds start to regrow and develop new leaves and stems in the early spring, and its canopy matures rapidly [70,71]. This species took about two months from the development of its first leaf to create full canopy maturity [64]. Although *P. montana* is a perennial plant species and 15-year-old stands have been found [64,72], information on the longevity of this species has not been made available.

*P. montana* grows well in full sunlight conditions, and its light compensation point of photosynthesis was 43 μmol m^−2^ s^−1^ [72,73]. However, the photosynthetic rate of this species is not high compared to that of other C_3_ plants. Its maximum photosynthesis rate was determined to be 23 μmol m^−2^ s^−1^ under field conditions [70]. The net carbon dioxide assimilation was between 11 and 27 μmol m^−2^ s^−1^, and its rate was similar to that of soybean [66,74]. The thick canopy layers and leaf reorientation of *P. montana* as described above may be able to maximize light perception and compensate for the carbon assimilation rate to maintain its growth and development.

These observations suggest that *P. montana* grows quickly, and forms thick canopy layers with highly branching stems and a great number of large leaves. This species effectively utilizes solar radiation for photosynthesis with the thick canopy layers and leaf movement, and allocates large amounts of photosynthetic fixed carbon to produce a great number of leaves. 

### 3.2. Reproduction

*P. montana* also allocates large amounts of photosynthetic fixed carbon for the generation of root systems. When the stems lie down on the soil surface, the nodes of the stems easily generate nodal roots [6,61]. About 25–45% of stem nodes occurred in the nodal roots, and 61 rooted nodes per m^2^ were counted [75]. The rooted nodes frequently detach from the mother plants within 1–3 years, and develop physiologically independent clonal plants defined as ramets [75]. The rooted nodes from the primary-, secondary- and higher-order branches lead to a high density of the independent ramets up to tens of thousands per hectare [14]. The ability to create a high density of ramets may work for this species’ reproduction. The dispersion of the rooted nodes from the population periphery via hurricanes and human activity may contribute the dispersal. The clonal rate of the 87 populations of *P. montana* in North America was recorded to be 80% [76].

*P. montana* develops flowers in mid- to late-summer in both hemispheres, and its fruits mature in the autumn. This species produces a low number of viable seeds relative to the number of flowers [16]. The possible reasons for the low number of viable seeds were considered to be the high degree of floral abscission within two days before and after flowering, and a small number of healthy seeds (5–30%) [16,77]. In addition, not all populations of *P. montana* flowered and produced visible seeds [16,17]. Only 6 of the 78 populations were recorded to produce viable seeds in USA [78]. The dispersion of the seeds occurred up to 25 m from the mother plants, but the majority of the seeds stayed within 6 m from the plants [77]. The seeds are possibly carried for long distances by water streams such as flood water.

The seed coats are very hard and impervious, and the scarification of the seeds increased the germination. The germination rate of the healthy seeds was 7–17% and 95–100% for the seeds without and with scarification, respectively [16,79]. The seeds were released from physical dormancy, which was caused by the seed coats, through mechanical scarification and the use of fire [16,80]. The changing temperatures, which ranged from 15 °C and 6 °C (day and night) to 35 °C and 15 °C (day and night), also increased the germination [79], which may indicate that the germination increased with the temperature increase from spring to summer. No seedlings emerged when the seeds were exposed to flooding for more than 7 days [79]. Only less than 10% of the seeds were reported to germinate and establish the seedling stage due to fungal diseases and insect predation under field conditions [77]. Since the seeds were covered by hard and impervious coats, the seeds can establish a seed bank. However, there has been no information made available on the longevity of *P. montana* seeds in the soil. 

These observations indicate that *P. montana* produces a small seed set and few seedlings, while it creates a very high density of clonal plants. Therefore, vegetative reproduction may be a more frequent reproduction strategy than sexual reproduction. 

### 3.3. Adaptation

*P. montana* thrives in areas such as forest margins, shrub areas, hill slopes, banks of water bodies, along the roads and railways, agricultural fields and disturbed lands [3,4,9,11,22]. This species was recorded to grow in mountainous areas up to 1200 in Japan and 1500 m in China [81,82]. It is also abundant in small islands, which have ecosystems that are very vulnerable to alien species [14,83]. This species grows well on fertile well-drained deep loamy soils with weak acidic (pH 4.5) to neutral (pH 7.0) conditions, but it can grow on many types of soils, including sandy and clay soils with a pH ranging from 3 to 8, and shallow and nutrient poor soils [16,22,23,82,84]. A total of 95% of fixed nitrogen via symbiosis with rhizobia was estimated to be supplied to the leaves when *P. montana* was grown in poor soil conditions [41]. Thus, the nitrogen-fixing ability of this species may contribute to its growth in poor soils. This species required an annual precipitation of 1000–15,000 mm for optimal growth [14]. It also grows well in irrigated areas such as agricultural fields where rainfall is less than 500 mm [83]. It can withstand relatively dry climate conditions because of reserved water in the large roots, which was described in the Introduction [14,83].

This species grows well in the areas with a hot summer (over 25 °C) and mild winter (5–15 °C) [14]. The average mean temperature of the northern limit of this species was 7 °C in Japan (native range) [17]. The northward distribution of this species was thought to be limited by cold temperatures [70,85]. Its large leaves die back at the first frost in winter season, and regrow from the stem nodes in early spring [1]. The above-ground stems of this species survived at −26 °C in the North America [70,86]. This species also regrows from under-ground stems, and snow protects the under-ground stems from lethal temperatures [70,86]. In fact, *P. montana* has already infested Benzie County (northwest Michigan, USA), where a temperature of below −20 °C has been recorded 13 times in the past 30 years [87,88]. This species was also found in southern Ontario, Canada, where the coldest time of year recorded ranged from −26 °C to −29 °C [2,86]. 

A high degree of genetic diversity of the *P. montana* population in USA was found [76,89,90,91]. Its high genetic diversity may reflect the history of this species introduced into the USA. Multiple introductions occurred over a long time from different origins of the native ranges, and were followed by genetic exchanges among populations [1,76]. However, the genetic diversity of the species in the native ranges was within the average of that of the herbaceous perennial plant species [92,93]. Plant species with a high genetic diversity showed better potential for adaptation to various environmental conditions [94,95].

These observations suggest that the adaptation of *P. montana* to various environmental conditions such as soil fertility, soil types, soil pH, annual precipitation and temperature is high. Its genetic diversity in the introduced range is also high. 

### 3.4. Allelopathy

The interaction of the alien plants with the indigenous plant species is one of the essential factors in the naturalization of alien plants in the introduced ranges [96,97,98,99]. Many invasive plants were reported to have an ability to perform allelopathy, which is the chemical interaction between donor plants and receiver plants [97,98,99,100]. Chemicals involved in allelopathy were defined as allelochemicals [100,101,102,103]. The allelochemicals are released into the neighboring environments including the rhizosphere soil from the donor plants through the rainfall leachates, volatilization, root exudation, and decomposition processes of donor plant residues. The allelochemicals are able to suppress the germination, growth and fitness of the neighboring plant species, and/or their mutualism with arbuscular mycorrhizal fungi and rhizobia [104,105,106,107,108]. Plants synthesize and store allelochemicals in some plant tissues until they release them into the neighboring environments [100,101,102,103]. Therefore, several researchers investigated the allelopathic activity in the extracts from different plant parts, the residues or litter of *P. montana*, and its rhizosphere soil.

Aqueous and methanol extracts of the leaves and roots of *P. montana* suppressed the germination and growth of *Lactuca sativa* L. and *Raphanus sativa* L., and the rhizosphere soil and litter of the species themselves suppressed the growth of *Raphanus sativa* and *Lolium perenne* L. Aqueous extracts of the litter of *P. montana* also suppressed the germination of *Bidens pilosa* L. and *Lolium perenne*. The pure soil mixed with the extracts of *P. montana* inhibited the root and shoot growth of *Bidens pilosa* and *Lolium perenne*. The total phenolic concentration in the *P. montana-*infested soil was 30- to 50-fold greater than that in the non-infested soil [109,110]. The investigations suggest that these phenolics may be involved in the inhibition caused by the litter and soils of *P. montana*. However, the chemical constituent of the phenolics has not been identified. The aqueous extracts of leaves, stems and roots of *P. montana* were also reported to suppress the germination of *Taraxacum officinale F.H.Wigg*, *Lolium multiflorum* Lam and *Echinochloa crus-galli* (L.) P.Beauv. [111].

**Figure 4:**
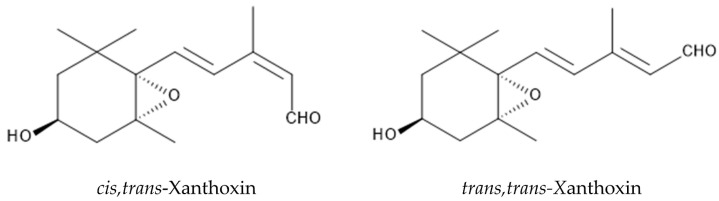
Possible allelochemicals in *P. montana*.

The sterilized quart sand mixed with the leaf powder of *P. montana* inhibited the germination, and the root and shoot growth of *Lepidium sativum* L., *Lactuca sativa*, *Phleum pratense* L. *Lolium multiflorum* [112]. Two allelopathic active substances were then isolated form the leaves of *P. montana,* and identified as *cis,trans*-xanthoxin and *trans,trans-*xanthoxin. The concentration of *cis,trans*-xanthoxin and *trans,trans-*xanthoxin was 51 ng and 73 ng per g leaf fresh weight, respectively. *cis,trans*-Xanthoxin and *trans,trans-*xanthoxin inhibited the growth of *Lepidium sativum* at concentrations greater than 0.3 μM and 3 μM, respectively. The concentration required for causing a 50% growth inhibition was 1.1 μM and 14 μM for *cis,trans*-xanthoxin and *trans,trans-*xanthoxin, respectively [113]. *cis,trans*-Xanthoxin was converted to abscisic acid (plant hormone) in some plants and cell-free systems [114,115,116,117]. Although the concentration of *trans,trans-*xanthoxin in plants was always greater than that of *cis,trans*-xanthoxin [113,118], *trans,trans*-xanthoxin was not converted to abscisic acid [119]. Both *cis,trans*-xanthoxin and *trans,trans-*xanthoxin themselves showed growth inhibitory activity on several plant species [118,120], which indicates that both xanthoxins may function in some physiological processes in these plants. Therefore, *cis,trans*-xanthoxin and *trans,trans-*xanthoxin may also be involved in the allelopathy of *P. montana* (Figure 4).

**Figure 5:**
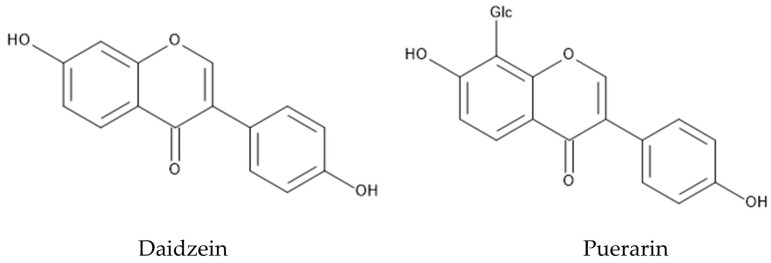
Major isoflavones in *P. montana*.

When the protoplasts of *P. montana* and *Lactuca sativa* obtained from their cotyledons were incubated together, the growth of *Lactuca sativa* protoplasts was inhibited by *P. montana* protoplasts in a protoplast-concentration-dependent manner [121]. Daidzein, which is one of the major isoflavones in the leaves of *P. montana* [122,123], disturbed the cell wall formation and cell division of *Lactuca sativa* protoplasts [121]. Therefore, the protoplast cells of *P. montana* may secrete daidzein into the growth mediums, and the secreted daidzein inhibits the growth of *Lactuca sativa* protoplasts as an allelochemical. Daidzein was also reported to be active in several pharmacological aspects [124] (Figure 5).

**Figure 6:**
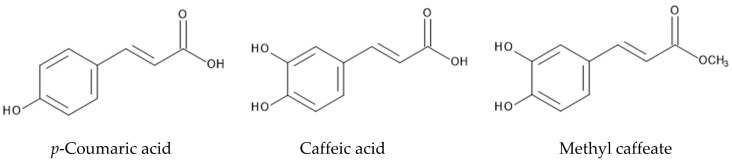
Phenylpropanoids in *P. montana,* having allelopathic activity.

Phenylpropanoids such as *p*-coumaric acid, caffeic acid and methyl caffeate were identified in the roots of *P. montana* [125]. *p*-Coumaric acid and caffeic acid were found in several other plant species, and showed germination and growth inhibitory activity as allelopathic agents [126,127,128]. Caffeic acid suppressed the germination and growth of target plant species due to the disturbance of water transport and photosynthesis, and the induction of IAA-oxidation [129,130]. *p*-Coumaric acid also disturbed water transport, and reduced the contents of chlorophyll A and B, and photosynthesis [130,131]. Methyl caffeate significantly inhibited the root and shoot growth of *Lepidium sativum* [132]. However, its mode of action has not been well documented (Figure 6).

These investigations suggest that leaves, roots and stems may contain water- and methanol-extractable allelochemicals, that some of them may be released from the residues or litter of this species into its rhizosphere soil, and that the soil may also contain some allelochemicals. *cis,trans*-Xanthoxin, *trans,trans-*xanthoxin, and daidzein were found in the leaves of *P. montana*, and may be involved in the allelopathy of *P. montana*. According to the novel weapons hypothesis, the competitive ability of invasive plants against indigenous plants is high due to the allelochemicals (weapons). These allelochemicals are released from the invasive plants, and enable the suppression of the germination, growth and regeneration of the indigenous plant species. These allelochemicals released from invasive plants are new to the indigenous plant species, because these indigenous plant species lack the co-evolutional history with the invasive plant species, and there has been no opportunity to develop tolerance towards these allelochemicals [96,97,133]. Therefore, these allelochemicals are effective in the inhibition of the indigenous plant species’ regeneration process in the introduced ranges, and contribute to the invasion.

### 3.5. Insecticidal and Fungicidal Activity

**Figure 7:**
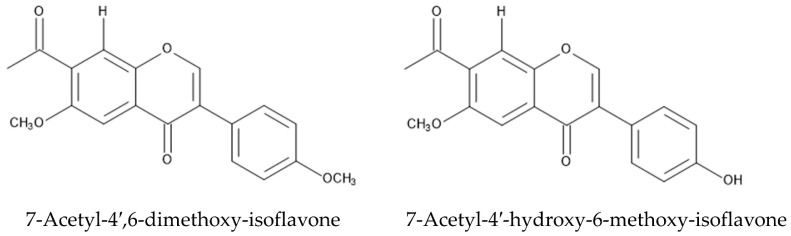
Isoflavones in *P. montana* with anti-virus activity.

The interaction between invasive plants and their natural enemies, such as herbivore insects and pathogens, is one of the important factors in the naturalization of invasive plants in the introduced ranges [98,134,135,136]. A moth species, soybean looper *Pseudoplusia includens* (Waker), was evaluated as a biocontrol agent for *P. montana.* However, the moths fed on *P. montana* showed a higher mortality and lower pupal weight than those fed on soybean [137], which suggests that certain compounds in the *P. montana* may be involved in the higher mortality and the lower pupal weight of the moths. The aqueous extract of *P. montana* suppressed the growth of the pathogenic fugus *Colletotrichum lagenarium* (Passerini) Ellis & Halsted, which was inoculated on the cotyledons and leaves of *Cucumis sativus* L. [138]. Two isoflavones, 7-acetyl-4′,6-dimethoxy-isoflavone and 7-acetyl-4′-hydroxy-6-methoxy-isoflavone, were isolated from *P. montana* and showed anti-tobacco mosaic virus activity [139]. These observations suggest that some compounds, including these isoflavones in *P. montana,* may work for anti-virus activity, and contribute to the fitness of *P. montana* into the introduced ranges (Figure 7).

Endophytes are present on the inside of plant tissues, and are involved in diverse and indispensable functions in plant growth, development, stress tolerance, and adaptation [140,141]. The interaction between endophytes and plants is species-specific, and most fungal endophyte species of *P. montana* belong to Ascomycota, Dothidemyceyes, Teremellales and Mycosphaerellaceae [142]. Some fungal endophytes in *P. montana* are involved in the growth suppression of pathogenic fungi such as *Fusarium oxysporum* [143]. Suppression was considered to be caused by the secretion of some secondary metabolites from the endophytes. These secondary metabolites suppressed the growth of the fungi as mycotoxins [144,145,146].

These investigations suggest that *P. montana* has anti-insecticidal and anti-virus activity and certain compounds, including isoflavones, may be involved in the activity. Endophytes in this species also suppress the growth of pathogenic fungi through mycotoxins. A great number of natural enemies such as herbivore insects and pathogens have been identified in *P. montana* stands in the native ranges in Japan and China [82,147,148]. However, fewer herbivore insects were found in the introduced ranges [11,22,149,150]. The condition of the existence of a few natural enemies may contribute to the superior growth rate and naturalization of *P. montana* in the introduced ranges.

### 3.6. Secondary Metabolites

**Figure 8:**
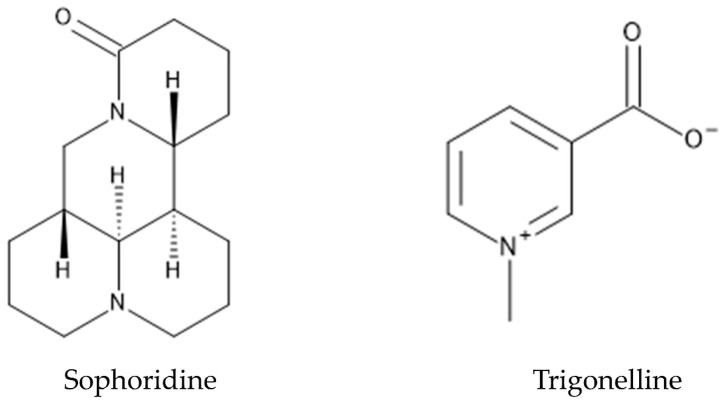
Alkaloids in *P. montana*.

*P. montana* is one of the most popular medicinal plants in Eastern Asia, and the dry roots and flowers of this species have been used in treatments of diabetes, fever, emesis, cardiac dysfunction and toxicosis [151,152,153]. Pharmacological investigations showed that the roots and flowers of *P. montana* contain a hundred polyphenolic compounds such as isoflavones, isoflavonoid glycosides, and saponins [154]. Isoflavones and their glycosides are the major pharmacological active constituents of these polyphenolic compounds, and puerarin and daidzein, among isoflavones and their glycosides, have been extensively investigated [155,156] (Figure 5). Puerarin is the most abundant secondary metabolite in the roots of *P. montana,* and showed a wide spectrum of pharmacological properties such as anti-diabetic activity, anti-inflammatory activity, anti-Parkinson’s disease activity, anti-Alzheimer’s disease activity, anti-isosteoporotic activity, and anti-cancer activity [155]. Daidzein was originally found in soybeans, and showed anti-diabetic activity and anti-inflammatory activity [157]. Alkaloids such as sophoridine and trigonelline were also found in the roots of *P. montana* [125]. Sophoridine showed anti-cancer, anti-inflammatory, and anti-bacterial activity [158]. Trigonelline was reported to show anti-Alzheimer’s disease activity and anti-diabetic activity [159] (Figure 8).

Although the majority of the identified secondary metabolites in *P. montana* have not yet been connected to the invasiveness of the plant species, some of these compounds may be involved in the allelopathy and defense functions against herbivores and pathogens. In fact, the extracts of the roots, leaves and rhizosphere soil of *P. montana* showed inhibitory activity on the germination and growth of several plant species and insecticidal activity, as described in the above section. Plants contain a large number of secondary metabolites in several chemical classes. The biosynthesis of certain secondary metabolites is increased or synthesized de novo under specific conditions [100,160,161,162,163,164]. Some of these secondary metabolites in invasive plants have been reported to show multiple functions such as allelopathic, anti-fungal, anti-microbial and anti-herbivore activity, and contribute to the fitness of the plants in the introduced ranges [97,99,165,166,167,168,169]. Therefore, some of the identified compounds and/or unidentified compounds in *P. montana* may contribute to the invasiveness and naturalization of *P. montana* in the introduced ranges. The possible invasive mechanisms of *P. montana* are summarized in Table 1.

## 4. Prospect

*P. montana* has not yet occupied all suitable climatic habitats in North America and other continents [170,171,172,173]. This species may possibly expand into those areas in the near further, and may cause serious damages to the economy and natural ecosystems. The global warming trends indicate an increase in the temperature and a decrease in the number of frost days [174,175,176]. The warming trends may favor the spread of *P. montana* northward in the northern hemisphere, and to higher altitudes because one of the limitation factors of its species distribution is cold temperatures [70,85]. The elevated CO_2_ level also enhanced the production and expansion of the species’ leaves, resulting in an increase in plant growth [177]. Modeling studies predicted the expansion of suitable climate conditions for the growth of *P. montana* in Canada, the USA, Switzerland, Italy, Austria and Slovenia [170,175,178].

The mechanical, chemical and biological approaches to control *P. montana* were well documented in the review articles [1,2]. The control of *P. montana* can be achieved with several herbicides such as glyphosate, picloram and triclopyr. However, the application of herbicide needs to be repeated for up to 10 years [179]. In addition, deeply buried roots and stems can escape herbicide application, and continue to support plant regrowth [1,2]. Many possible biological control agents were selected in the native ranges, and some of them showed significant effects in the control of *P. montana* [84,180]. However, this seems to still be under experimental stages [2,11]. Young small stands of *P. montana* can be eliminate in several years through mowing, grazing and burning [14]. However, all roots need to be killed or removed to prevent plant regrowth [1,2]. Therefore, the control of *P. montana* seems to be very difficult. However, the eradication of *P. montana* stands may be able to be accomplished through integrated management and continuous treatments [181]. An early detection and rapid response, education and awareness program and any efforts would help to prevent the future spread of *P. montana* as described in [182].

## 5. Conclusions

*P. montana* infestation reduced the diversity and number of indigenous plant species, and altered the species constitution of the arbuscular mycorrhizal fungi in the introduced ranges. This species also affected the concentration of carbon and nitrogen in the soil and aquatic environments, and increased the amount of pollutants in the local atmosphere. Its infestation also caused serious economic losses in forestry and agriculture.

*P. montana* grows quickly, and forms the thick canopy layers with highly branching stems and a great number of large leaves. This species effectively utilizes solar radiation for photosynthesis with thick canopy layers and leaf reorientation, and allocates large amounts of photosynthetic fixed carbon to support the canopy structure. The thick canopy structure of this species prevents sunlight from penetrating into under-growth vegetation and trees covered with its large leaves, killing those plant species. This species allocates large amounts of photosynthetic fixed carbon to create extensive root systems, and the stems easily generate nodal roots. The rooted nodes frequently detach from the mother plants and develop clonal plants. Tens of thousands of independent clonal plants per hectare were counted. The adaptation ability of this species is high, and it grows under various environmental conditions of soil fertility, soil types, soil pH, annual precipitation and temperature. Its characteristics of fast growth, thick canopy structure, high rate of the vegetative reproduction, and adaptative ability to various environmental conditions may contribute to the invasiveness of this species.

*P. montana* produces various secondary metabolites involved in defense functions against natural enemies such as herbivores, and pathogenic fungi and viruses. The allelopathy of this species may cause the suppression of the germination and growth, and the disturbance of the regeneration process of indigenous plant species through the release of allelochemicals. The characteristics of *P. montana* for defense functions and allelopathy may also contribute to the invasiveness of this species. In addition, a great number of herbivore insects and pathogenic fungi have been identified in *P. montana* stands in the native ranges, whereas fewer herbivore insects have been found in the introduced ranges. The existence of a few natural enemies may contribute to the superior growth rate and naturalization of *P. montana* in the introduced ranges. The global warming trends may favor the spread of this species into additional non-native areas, and may increase the threat of this species.

## Figures and Tables

**Table 1:** Possible invasive mechanisms of *P. montana*.

Characteristic	Reference
➢ Grows rapidly and elongates 20–30 m long per single growing season.	[6,59,60,61]
➢ Forms thick copy layers with highly branched stems and a great number of large leaves.	[5,64,65,66,67]
➢ Creates a very high density of clonal plants up to tens of thousands per hectare.	[6,14,61,75,76]
➢ High adaptative ability to soil fertility, soil types, soil pH, annual precipitation and temperature.	[14,16,17,22,23,41,70,81,82,83,84,86]
➢ Allelopathy and allelochemicals, suppression of the germination, growth and regeneration of the indigenous plant species.	[109,110,111,112,113,121,122,123,125]
➢ Defense function against herbivore insects and pathogens. A few natural enemies in the introduced ranges.	[82,137,138,139,142,143,147,148,149,150]
➢ Secondary metabolite.	[125,154,155,156,157]

## Data Availability

Not applicable.
